# Auranofin and Cold Atmospheric Plasma Synergize to Trigger Distinct Cell Death Mechanisms and Immunogenic Responses in Glioblastoma

**DOI:** 10.3390/cells10112936

**Published:** 2021-10-28

**Authors:** Jinthe Van Loenhout, Laurie Freire Boullosa, Delphine Quatannens, Jorrit De Waele, Céline Merlin, Hilde Lambrechts, Ho Wa Lau, Christophe Hermans, Abraham Lin, Filip Lardon, Marc Peeters, Annemie Bogaerts, Evelien Smits, Christophe Deben

**Affiliations:** 1 Center for Oncological Research (CORE), Integrated Personalized & Precision Oncology Network (IPPON), University of Antwerp, 2610 Wilrijk, Belgium; Jinthe.VanLoenhout@uantwerpen.be (J.V.L.); Laurie.Freireboullosa@uantwerpen.be (L.F.B.); Delphine.Quatannens@uantwerpen.be (D.Q.); Jorrit.DeWaele@uantwerpen.be (J.D.W.); Celine.Merlin@uantwerpen.be (C.M.); Hilde.Lambrechts@uantwerpen.be (H.L.); Howa.Lau@uantwerpen.be (H.W.L.); Christophe.Hermans@uantwerpen.be (C.H.); Abraham.Lin@uantwerpen.be (A.L.); Filip.Lardon@uantwerpen.be (F.L.); Marc.Peeters@uantwerpen.be (M.P.); Evelien.Smits@uza.be (E.S.); 2 Plasma Lab for Applications in Sustainability and Medicine ANTwerp (PLASMANT), University of Antwerp, 2610 Wilrijk, Belgium; annemie.bogaerts@uantwerpen.be; 3 Department of Oncology, Multidisciplinary Oncological Center Antwerp, Antwerp University Hospital, 2650 Edegem, Belgium

**Keywords:** oxidative stress, auranofin, cold atmospheric plasma, glioblastoma, cancer cell death

## Abstract

Targeting the redox balance of malignant cells via the delivery of high oxidative stress unlocks a potential therapeutic strategy against glioblastoma (GBM). We investigated a novel reactive oxygen species (ROS)-inducing combination treatment strategy, by increasing exogenous ROS via cold atmospheric plasma and inhibiting the endogenous protective antioxidant system via auranofin (AF), a thioredoxin reductase 1 (TrxR) inhibitor. The sequential combination treatment of AF and cold atmospheric plasma-treated PBS (pPBS), or AF and direct plasma application, resulted in a synergistic response in 2D and 3D GBM cell cultures, respectively. Differences in the baseline protein levels related to the antioxidant systems explained the cell-line-dependent sensitivity towards the combination treatment. The highest decrease of TrxR activity and GSH levels was observed after combination treatment of AF and pPBS when compared to AF and pPBS monotherapies. This combination also led to the highest accumulation of intracellular ROS. We confirmed a ROS-mediated response to the combination of AF and pPBS, which was able to induce distinct cell death mechanisms. On the one hand, an increase in caspase-3/7 activity, with an increase in the proportion of annexin V positive cells, indicates the induction of apoptosis in the GBM cells. On the other hand, lipid peroxidation and inhibition of cell death through an iron chelator suggest the involvement of ferroptosis in the GBM cell lines. Both cell death mechanisms induced by the combination of AF and pPBS resulted in a significant increase in danger signals (ecto-calreticulin, ATP and HMGB1) and dendritic cell maturation, indicating a potential increase in immunogenicity, although the phagocytotic capacity of dendritic cells was inhibited by AF. In vivo, sequential combination treatment of AF and cold atmospheric plasma both reduced tumor growth kinetics and prolonged survival in GBM-bearing mice. Thus, our study provides a novel therapeutic strategy for GBM to enhance the efficacy of oxidative stress-inducing therapy through a combination of AF and cold atmospheric plasma.

## 1. Introduction

Glioblastoma (GBM) is the most prevalent malignant, primary brain tumor, which carries an extremely poor prognosis due to its aggressive and invasive nature [1]. Despite current improvements in conventional treatment, tumor recurrence is nearly inevitable, contributing to a median survival duration of only 14.6 months and a five-year survival rate of less than 5.6% [2,3]. Therefore, the development of new treatment strategies is urgently required. 

Malignant cells are characterized by higher levels of intrinsic reactive oxygen species (ROS) when compared to normal cells as a consequence of, for example, altered metabolic rate and gene mutations [4]. To maintain redox balance, malignant cells counter this intrinsic oxidative stress by upregulation of their antioxidant defense system [5]. The difference in redox balance between malignant cells and normal cells unlocks a potential therapeutic strategy for ROS-inducing therapies [6]. 

We investigated a novel combinatory therapeutic strategy, by inducing high oxidative stress through the delivery of exogenous ROS and the inhibition of endogenous, protective, antioxidant systems. We hypothesized that the combination of these two different ROS-modulating methods would be a beneficial and promising anti-GBM treatment strategy. Firstly, cold atmospheric plasma was used as a unique treatment method for increasing oxidative stress levels to target cancer cells via the exogenous delivery of ROS and reactive nitrogen species (RNS) [7,8]. This cold atmospheric plasma is an ionized gas that is composed of ROS and RNS, excited molecules, ions, electrons and other physical factors, such as electromagnetic fields and ultraviolet radiation [9]. This plasma can be delivered directly onto the tumor, or indirectly through plasma-treated liquids [10]. This type of exogenous ROS-inducing therapy has already been investigated in different cancer types in vitro and in vivo, including GBM [10,11,12,13]. Secondly, the endogenous induction of oxidative stress was achieved by the inhibition of the antioxidant defense system using auranofin (AF), a thioredoxin reductase 1 (TrxR) inhibitor. AF forms a stable, coordinative bond between its gold(I) center and the active site of selenocysteine residues, which causes an increase in oxidative stress, as TrxR is an enzyme that catalyzes the reduction of Trx with electrons from NADPH in the Trx antioxidant system [14,15]. AF has also gained research interest over the past years, as a non-cancer drug for new repurposing into oncology by the Repurposing Drugs in Oncology project. Several advantages of AF include oral administration, lipophilic properties and approval of the organogold compound by the Food and Drug Administration (FDA) for the treatment of rheumatoid arthritis [16]. In GBM, AF is one of nine drugs in the CUSP9 treatment protocol which uses re-purposed, older drugs and is currently undergoing a clinical trial (NCT02770378) as an add-on treatment to the standard-of-care, temozolomide, for recurrent GBM [17]. Here, AF is also used as one of nine drugs in the combination strategy to increase ROS-mediated cell death, highlighting the potential of AF in GBM research [18]. 

Targeting malignant cells via different sources of ROS could be a promising novel treatment strategy for GBM. Therefore, we studied the cellular response upon combination treatment of AF with cold atmospheric plasma to determine whether this combination enhanced the cytotoxic effect in 2D and 3D GBM cell cultures. In addition, we performed an in-depth analysis of the molecular mechanisms underlying the responses of different GBM cell lines to the combination of AF and cold atmospheric plasma-treated PBS (pPBS). This study is the first to show that the response to AF is synergistically enhanced by sequential addition of pPBS or by direct plasma treatment in both 2D and 3D cell cultures, respectively. This combination was able to deliver high amounts of ROS and induced distinct characteristics which indicated underlying mechanisms of cell death, including apoptosis and ferroptosis. Additionally, we showed that these dying cancer cells were able to initiate the release of immunogenic cell-death (ICD) related damage-associated molecular patterns (DAMPs) and subsequently enhanced dendritic cell (DC) maturation. Contrary to these immunostimulatory effects, we also found that the phagocytotic capacity of the DCs was inhibited by AF. In vivo, AF in combination with cold atmospheric plasma effectively reduced tumor growth and prolonged survival.

## 2. Materials and Methods

### 2.1. Cell Lines and Cell Culture

The human GBM cell lines U-87 MG (kindly provided by Dr. Margaret Ashcroft, University of Cambridge), LN-229 (ATCC CRL-261) and T98G (kindly provided by Dr. Nicolas Goffart, University of Liège) were cultured in Dulbecco’s Modified Eagle Medium (DMEM, Life Technologies) supplemented with 10% fetal bovine serum (FBS, Life Technologies), 1% penicillin/streptomycin (Life Technologies) and 2 mM L-glutamine (Life Technologies). Cells were maintained in exponential growth phase at 5% CO_2_ in a humidified incubator at 37 °C. T98G cells had green autofluorescence and could therefore not be considered for certain experiments. To rule out differences in sensitivity to AF due to variations in selenium concentrations [19,20] all experiments were performed using the same supplier of FBS, and triplicates of TrxR activity and GSH content were performed with the same batch of treated cells and growth medium. Cell cultures were tested regularly for the absence of mycoplasma contamination using the MycoAlert detection kit (Lonza). For some experiments, cells were transduced with the IncyCyte® Nuclight Red Lentivirus reagent (Essen Biosciences) using the manufacturer’s protocol. 

### 2.2. Generation of Spheroids

Cell suspensions were prepared at 5 × 104 cells/mL for U-87, 6 × 104 cells/mL for T98G and 7 × 104 cells/mL for LN-229. Different concentrations of different cell lines were used to maintain a diameter of approximately 500µm. Cells were seeded in an ultra-low attachment (ULA) 96-well plate (round bottom, Corning Costar) in DMEM and centrifuged for 10 min at 100× g. Spheroids were allowed to form and grow for 3 days at 5% CO_2_ in a humidified incubator at 37 °C prior to their use in experiments. 

### 2.3. Treatment of 2D and 3D Cell Cultures

Cells were incubated with AF (0–10 µM, Bio-Techne) as single agents for different time periods, according to the experiment. 

Two-dimensional cell cultures were treated indirectly with pPBS which was generated using an atmospheric pressure plasma jet kINPenIND^®^ (Neoplas Tools), as previously used in our lab and described by Van Loenhout et al. [12]. Argon gas was used in this setting as feeding gas [21]. Then, 2 mL of PBS was treated with one standard liter per minute (slm) gas flow rate at a gap distance of 6 mm for 5 min. This 100% plasma-treated PBS (pPBS) was further diluted into PBS to final concentrations of 25, 50, and 62.5% pPBS, which was then directly added in a 1/6 dilution into the media of the cells. Under these conditions, the 100% pPBS contained 526.91 µM H_2_O_2_, 56.27 µM NO_2_^−^, and 37.75 µM NO_3^−^_ and these values were determined using a fluorometric assay for H_2_O_2_ (Sigma-Aldrich) and a colorimetric assay for NO_2_^−^ and NO_3_^−^ (Cayman chemicals), according to manufacturer’s instructions. These concentrations were 4-fold diluted in the 25% pPBS treatment conditions which contained 128.46 µM H_2_O_2_, 14.91 µM NO_2_^−^, and 14.41 µM NO_3_^−^. Untreated PBS was used as vehicle control for all experiments. 

Three-dimensional cell cultures were treated using the COST jet plasma setup, as previously optimized in our lab and described by Privat-Maldonado et al. [22]. It was operated with a feed gas of He with 5% H_2_O vapor mixture which was achieved using a split He flow, by passing part of it through an H_2_O-filled Drechsel flask. Before treatment, 3-day old spheroids were washed once with PBS after removing the culture medium. Direct treatments were performed on spheroids in 200 µL of PBS in a 96-well ULA plate for 3 min. Spheroids in 200 µL of untreated PBS were used as vehicle controls. Under these conditions, the pPBS contained 1230.82 µM H_2_O_2_, 4.98 µM NO_2_^−^, and 5.03 µM NO_3_^−^ [22]. Spheroids were incubated for 90 min with the treatment, after which it was replaced with the supernatant of the corresponding spheroid. 

For combination treatments, the 2D or 3D cell cultures were pretreated for 4 h with AF (0–10 µM). Afterwards, the 2D and 3D cell cultures were treated with pPBS and plasma, respectively, as described. 

### 2.4. Cell Death Assays and Synergism

For the 2D cell cultures, cell death was determined using the IncuCyte ZOOM^®^ Live-Cell Imaging System (Sartorius). All experiments were performed at least three independent times. NucLight red lentiviral-transduced GBM cell lines were seeded at a density of 2 × 104 cells/mL in a 96-well pate. After overnight incubation, cells were treated with mono- and/or combination treatment of AF (0–7.5 µM) and pPBS (25, 50, and 62.5%), in the presence of IncuCyte^®^ Cytotox Green reagent (50 nM, Essen BioScience). Treatment of the cells was done in the absence or presence of the desired cell death inhibitors, with a preincubation of 1 h for n-acetylcysteine (NAC, 5 mM, Sigma-Aldrich), catalase (20 µg/mL, Sigma-Aldrich) ferrostatin-1 (Fer-1, 1 µM, Sigma-Aldrich) and a preincubation for 4 h with deferoxamine (DFO, 100 µM for LN-229 and T98G cells and 50 µM for U87 cells, Sigma-Aldrich). Plates were incubated in the temperature- and CO_2_-controlled IncuCyte^®^ Live-Cell Imaging System (Sartorius) for 72 h. Cell death was monitored by taking images every 24 h, to limit phototoxicity. For analysis, green object count (1/mm²), red object count (1/mm²) and green-red overlapping object count (1/mm²) were determined with the IncuCyte ZOOM^®^ software. The percentage of cell death was calculated using the formula: [green object count/((red object count + green object count)—overlapping object count)] *100. The percentage of survival was calculated using the red object count, which was normalized towards the untreated control. 

Caspase-3/7 activity was also determined using the IncuCyte ZOOM^®^ Live-Cell Analysis System in the presence of Caspase-3/7 Green apoptosis reagent (2.5 µM, Essen Bioscience). The percentage of caspase-3/7 positive cells was calculated using the formula: [green object count/((red object count + green object count)—overlapping object count)] *100.

For the 3D cell cultures, spheroids were treated with mono- and/or combination treatments of AF (0–10 µM) and plasma, using the COST jet device. Microscopic images were taken with the IncuCyte^®^ system at different time points. End-point viability of spheroids was assessed after 72 h using the CellTiterGlo^®^ 3D Cell Viability assay (Promega), according to the manufacturer’s protocol. The luminescent signal was measured using Spark^®^Cyto (Tecan). 

In order to determine the presence of a synergistic effect, the combination index (CI) was analyzed according to the Additive Model, which is determined based on the ratio value between the found and the expected combination effect, as calculated from the exposure of the individual treatments [23]. 

### 2.5. ROS Measurement

Cells were seeded in 96-well plates, incubated overnight and exposed to mono- and/or combination treatment of AF (0–7.5 µM) and plasma (25%, 50, and 62.5 pPBS). Immediately following treatment, 2.5 µM CellROX Green reagent (Invitrogen) was added to U-87 and LN-229 cells and 5 µM CellROX Red reagent (Invitrogen) was added to T98G cells. Afterwards, the plate was transferred to the temperature- and CO_2_-controlled IncuCyte ZOOM®. ROS was monitored over time by pictures that were taken at 4 and 24 h after treatment. For analysis, the average green calibrated unit (GCU) and average red calibrated unit (RCU) were plotted for every cell line after 4 and 24 h.

### 2.6. Protein Isolation

For protein-based experiments, cells were seeded and treated with mono- and/or combination treatment of AF and pPBS. After 4 h of the final treatment, cells were lysed in lysis buffer (10 mM TrisHCl, 400 mM NaCl, 1mM EDTA, 0.1% NP40, and protease inhibitor). After centrifugation (10 min, 13 000 rpm, 4 °C), cleared lysates that contained the isolated proteins were harvested and kept at −20 °C. Protein concentrations were determined using a Pierce BCA protein kit (Thermo Scientific), according to the manufacturer’s instructions. To determine the baseline protein levels, cells were collected after sub-culturing and lysed as described above.

### 2.7. Thioredoxin Reductase Activity Assay

The treated and untreated control protein lysates were used to measure TrxR activity, which was determined by using the Thioredoxin Reductase Colorimetric Assay Kit (Cayman Chemical), according to the manufacturer’s protocols. Absorbance was recorded at 405 nm with the Spark®Cyto (Tecan) during the initial 5 min of the reaction. TrxR activity was calculated using the formula provided by the protocol, whereby background measurements were subtracted from all values. An equal amount of protein was loaded for each condition as determined by the Pierce BCA protein kit.

### 2.8. Glutathione Level Quantification

Cells were seeded in 96-well plates and treated with mono- and/or combination treatment of AF and pPBS. After 4 and 24 h of the last treatment, the cellular concentrations of glutathione (GSH) and oxidized glutathione (GSSG) were determined by using the GSH/GSSG-GloTM Assay kit (Promega), according to the manufacturer’s protocols. Luminescent intensity was measured using the Spark®Cyto (Tecan). The amount of GSH, proportional to the luminescent signal, was corrected for the number of cells present in the well. 

### 2.9. Lipid Peroxidation

Cellular lipid ROS was measured using the Image-iTTM Lipid Peroxidation Kit (Invitrogen), according to the manufacturer’s instructions. Therefore, U-87 and LN-229 were treated with mono- and/or combination treatment of AF (0–1.5–2 µM) and pPBS (25 and 50% pPBS) for 48 h, or the positive control (cumene hydroperoxide) for 2 h Afterwards, 10 µM of the C11-BODIPY dye was added to the culture and incubated for 30 min at 37 °C. The T98G cell line could not be included in this assay due to autofluorescence. Acquisition was performed on a CytoFLEX (BD), and FlowJo v10.1 software (TreeStar) was used to calculate the ratios of the C11-BODIPY red over green mean fluorescence intensity (MFI) signals.

### 2.10. Analysis of ICD-Related Markers

All the GBM cell lines were seeded and treated with mono- and/or combination treatment of AF and pPBS, and the analysis of ICD-related markers occurred at different time points. At 48 h after treatment, cells were stained for membrane (calreticulin) CRT expression. Here, cells were harvested and incubated with 5% normal goat serum (NGS, Sigma-Aldrich), followed by washing and incubation with an AF488-conjugated anti-CRT antibody (Abcam) for U-87 and LN-229 cell lines, and with an AF647-conjugated anti-CRT antibody (Abcam) for the T98G cell line (due to the autofluorescence of this cell line), for 40 min. Prior to analysis, the cells were stained with Annexin V (AnnV; BD) and propidium iodide (PI; BD) to distinguish between early apoptotic and necrotic cells. Cell debris and necrotic cells (PI+) were excluded from the analysis. For every sample, a corresponding isotype control was used (Abcam). Flow cytometric acquisition was performed on an AccuriTM C6 instrument (BD). Extracellular ATP release (nmol) was measured in conditioned media (supplemented with heat-inactivated FBS) 4 h after treatment via an ENLITEN^®^ ATP assay system, according to the manufacturer’s protocol (Promega). The bioluminescent signal was measured using a Spark^®^Cyto (Tecan, Männedorf, Switzerland) device. Release of HMGB1 (ng/mL) was analyzed 48 h after treatment using an enzyme-linked immunosorbent assay (ELISA, Tecan, Männedorf, Switzerland). The absorption was measured using an iMARK^TM^ plate reader (Bio-rad, Temse, Belgium).

### 2.11. In Vitro Generation of Human Monocyte-Derived Immature DC

Human peripheral blood mononuclear cells (PBMC) were isolated by LymphoPrep gradient separation (Sanbio, 1114547), from a buffy coat of healthy donors (Ethics Committee of the University of Antwerp, reference number 13/46/454) isolated from adult volunteer whole blood donations (supplied by the Red Cross Flanders Blood service, Belgium). Monocytes were isolated from PBMC using CD14 microbeads, according to the manufacturer’s protocol (Miltenyi, Biotec, Leiden, The Netherlands). The purity after isolation was >90%. After isolation, CD14+ cells were plated at a density of 1.25–1.35 × 106 cells per mL in 1640 RPMI, supplemented with 2.5% human AB (hAB, Sanbio) serum, 800 U/mL granulocyte-macrophage colony stimulating factor (GM-CSF; Gentaur, Kampenhout, Belgium) and 20 ng/mL interleukin (IL)-4 (Miltenyi, Biotec, Leiden, The Netherlands) at day 0, as described before [24]. Immature DCs were harvested on day 5.

### 2.12. Maturation Status and Phagocytotic Capacity of DCs

After the in vitro generation of DCs, GBM cell lines were labeled with the green-fluorescent-membrane dye PKH67 (Sigma Aldrich, Overijse, Belgium) and seeded in 6-well plates for overnight incubation. The labeling of tumor cells with PKH67 was carried out according to the manufacturer’s instructions. On day 5, tumor cells were pretreated with AF (0–7.5 µM). After 4 h of pretreatment, tumor cells were treated with pPBS (25, 50, and 62.5% pPBS). In order to make a distinction between the target and effector cells, immature DCs were also labeled with a fluorescent dye. Briefly, DCs were labeled with 2 µM of violet-fluorescent CellTracker Violet BMQC dye (Invitrogen, Bleiswijk, The Netherlands) at a concentration of 1 × 106 cells per mL at 37 °C. Four hours after pPBS treatment, effector and target cells were cocultured at a 1:1 effector:target (E:T) ratio. On day 7, cells were collected and used immediately for the flow cytometric detection of DC maturation markers and phagocytosis. Expression of anti-CD86-PECy7, anti-CD80-PerCP5.5, and anti-major histocompatibility complexes class II (MHC-II)-APC were measured on the Violet+ viable (Live/Dead Near IR+) DC population. For every marker, an isotype control was used to subtract aspecific signals. Results are represented as ΔMFI ((MFI staining treated–MFI isotype treated)–(MFI staining untreated–MFI isotype untreated)) and as the percentage of the DCs which were double positive for MHC-II and CD86. Phagocytosis of PKH67+ tumor cells by violet-labeled DCs is expressed as %PKH67+violet+ cells within the violet+ DC population. Acquisition was performed on a FACSAria II (BD). Data analysis was performed using FlowJo v10.1 software (BD, OR, USA).

### 2.13. Animal Experiment

Female C57BL/6J mice, age 6–10 weeks, were obtained from Jackson Laboratories and maintained at the animal core facility of the University of Antwerp. All animal procedures were conducted in accordance with approval of the Animal Ethics Committee of the University of Antwerp under registration number 2020-20. All mice were housed in filter-top cages which were enriched with houses and nesting material. Mice were checked on a daily basis to inspect their health and wellbeing. Mice were given at least a 7 day adaption period upon arrival before being included in experiments, to reduce stress levels. 

The SB28 cell line (provided by H. Okada, UCSF, San Francisco, CA, USA) was cultured in DMEM supplemented with 10% heat-inactivated FBS, 1% penicillin/streptomycin, 1% HEPES and 1% GlutaMAX and maintained at 37 °C and 5% CO2. The cell line was routinely tested for mycoplasma contamination. The cells were used in the experiments between passage three and six after thawing.

Mice were inoculated subcutaneously into the shaved abdominal flank with 1 × 10^6^ SB28 cells, suspended in 100 µL PBS. Tumor size was monitored with calipers and the tumor volume (mm³) was calculated using the formula (length × width²)/2. When tumors reached an average size of approximately 30 mm³, the mice were randomized based on their tumor size and divided over the different treatment groups. Tumor size was measured thrice a week. Mice were euthanized when a tumor size of 1500 mm³ was reached.

AF (15 mg/kg) was administered daily via oral gavage using a 20 G flexible feeding needle for a period of 14 days. 

A microsecond-pulsed dielectric barrier discharge (DBD) system which was previously described [7,25] was used for the CAP treatments. Briefly, a microsecond pulser (Megaimpulse Ltd., Russia) generated a 30 kV output pulse with the rise time fixed within 1–1.5 μs and a pulse width of 2 μs. The frequency of the pulses was fixed at 700 Hz with a treatment time of 10 s. Treatment was performed for 5 consecutive days. The applicator of the system was a copper electrode, covered with dielectric quartz, and was connected to the output of the microsecond pulser. The applicator was held by hand above the tumor (approximately 1–4 mm) for treatment. Here, an electrically safe plasma was created in direct contact with the tumor, and the surrounding gas and tissue were not significantly heated. During the treatment, mice were sedated using IsoFlo^®^ inhalation vapour. 

### 2.14. Statistical Analysis

All experiments were performed at least in triplicate. Prism 9.0 software (GraphPad) was used for data comparison and graphical data representations. All statistical analyses were performed in JMP Pro 15.1 and SPSS Statistics 27 software. The interaction term of AF and plasma was statistically analyzed using linear mixed models. The non-parametric Kruskal-Wallis test was used to compare means between more than two groups. The non-parametric Mann-Whitney U test was used to compare means between two groups. Statistical differences in tumor kinetics between the different treatment groups in the different experiments were determined using a linear mixed model analysis. Differences in survival were analyzed using a Log-rank test. *p*-values < 0.05 were considered statistically significant. 

## 3. Results

### 3.1. The Combination Treatment of AF and pPBS Leads to Synergistic Response in Cell Growth Inhibition and Cell Death in GBM Cell Lines 

**Figure 1:**
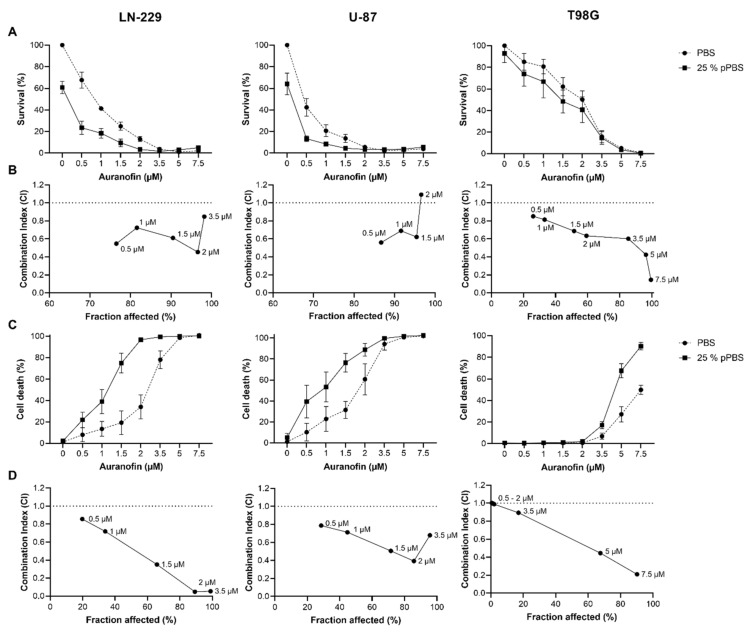
The sequential combination treatment of AF and pPBS induces a synergistic response in cell survival and cell death of GBM cell lines. (**A**) Dose–response survival curves after 72 h of AF (0–7.5 µM) monotherapy and treatment in combination with 25% pPBS. (**B**) The corresponding combination indexes (CI) for each AF concentration based on survival. (**C**) Dose–response curves of the cytotoxic effect after 72 h of AF (0–7.5 µM) monotherapy and treatment in combination with 25% pPBS. (**D**) The corresponding combination indexes for each AF concentration based on cytotoxicity. CI > 1 indicates an antagonistic effect, CI = 1 an additive effect and CI < 1 a synergistic effect. Fraction affected indicates the fraction of cells (in percentages) affected by AF. The supporting data and statistics for this figure can be found in Table 1. Graphs represent mean ± SEM of ≥3 independent experiments.

In order to investigate the potential interaction between AF and pPBS, 2D cell cultures of three different GBM cell lines (LN-229, U-87 and T98G) were incubated with an 8-point titration of 0–7.5 µM AF for 4 h, followed by treatment with PBS or 25% pPBS for a total of 72 h. The induction of cell death and growth inhibition were investigated to determine the EC50 and IC50 values, respectively. Dose–response survival and cytotoxicity curves (Figure 1a,b) show that all cell lines had distinct sensitivity towards mono- and combination treatments of AF and pPBS. In all cell lines, the IC50 values of both AF monotherapy treatment and treatment in combination with 25% pPBS were lower than the EC50 values, indicating that at lower concentrations cell growth was inhibited and at higher concentrations cell death was induced (Table 1). LN-229 (IC50 = 0.762 µM AF; EC50 = 2.335 µM AF) and U-87 (IC50 = 0.455 µM AF; EC50 = 1.739 µM AF) could be considered as sensitive cell lines when compared to T98G (IC50 = 2.364 µM AF; EC50 = 7.395 µM AF), which was not responsive to lower concentrations of AF, indicating that T98G is a more resistant cell line towards mono- and combination therapies of AF and pPBS. 

When cells were incubated with AF for 4 h before treatment with 25% pPBS, the cytotoxic effect of AF was amplified and led to a strong synergistic effect (combination index (CI) < 1, Figure 1c,d). The effects of AF and pPBS, as well as their interactions, on cell death and cell survival were statistically analyzed using linear mixed models. A significant interaction indicates that the effect of pPBS on cell death and cell survival is dependent on the concentration of AF, and vice versa. Table 1 gives a detailed overview of the synergistic effects of AF and pPBS on cell death and cell survival in all examined cell lines. Importantly, in the T98G cell line there was only a synergistic effect observed with higher concentration of AF. Therefore, we present the combination index for both cell survival and cell death based on higher AF concentrations (5 µM) when compared to U-87 and LN-229 cell lines (1.5 µM) in Table 1. The addition of pPBS to the AF treatment significantly enhanced cell death compared to AF and pPBS monotherapies in all cell lines. A significant interaction on the inhibition of cell survival was only seen for the sensitive cell lines LN-229 and U-87. These data show that the combination of AF and pPBS synergistically enhances the induction of GBM cell death in 2D cell cultures. 

### 3.2. The Combination Treatment of AF and pPBS Causes Alterations in Protein Targets Related to the Antioxidant Defense System

**Figure 2:**
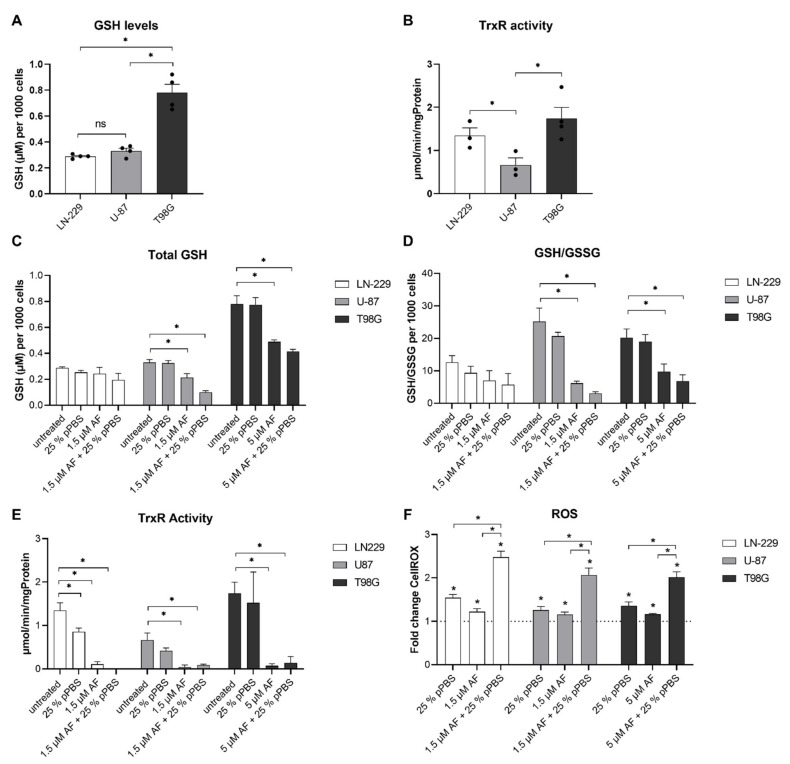
The effect of combination treatment of AF and pPBS on protein targets related to the antioxidant defense system. (**A**) Baseline GSH protein levels of LN-229, U-87 and T98G cells normalized towards 1000 cells (**B**) Baseline TrxR activity of LN-229, U-87 and T98G cells. (**C**) GSH protein levels normalized towards 1000 cells after 4 h of treatment with monotherapies and combination therapy of AF (1.5 µM or 5 µM) and 25% pPBS. (**D**) Ratio GSH/GSSG normalized towards 1000 cells after 4 h of treatment with monotherapies and the combination of AF (1.5 µM or 5 µM) and 25% pPBS. (**E**) TrxR activity after 4 h of treatment with monotherapies and combination therapy of AF (1.5 µM or 5 µM) and 25% pPBS. (**F**) Intracellular ROS levels shown as fold change of CellROX Calibration Units (CU), relative towards untreated after 4 h of treatment with monotherapies and combination therapy of AF (1.5 µM or 5 µM) and 25% pPBS. Graphs represent mean ± SEM of ≥3 independent experiments. * *p* ≤ 0.05 denotes a statistically significant difference compared with untreated control, ns denotes no statistical difference (*p* > 0.05).

To further investigate the observed difference in sensitivity between the GBM cell lines, the baseline expression levels of GSH and TrxR activity were determined (Figure 2a,b). Statistically, a significant difference between GSH levels of the sensitive cell lines (LN-229 and U-87) and GSH levels of the more resistant T98G was observed. Additionally, T98G cells showed the highest baseline TrxR activity when compared to the baseline levels of U-87 and LN-229 cell lines. The U-87 cell line showed the lowest baseline TrxR activity, which was significantly lower when compared to the two other cell lines (Figure 2b). 

Since T98G had the highest total GSH levels, and even with greater than three-fold higher concentrations of AF, residual GSH levels were still higher when compared to baseline GSH levels of U-87 and LN-229. Therefore, we concluded that this higher antioxidant buffering capacity offered by the GSH system could be an important factor in explaining the reduced sensitivity of T98G, when compared to U-87 and LN229, towards AF monotreatment and treatment in combination with pPBS. 

Next, alterations in key regulators of the ROS scavenging system were examined upon mono- and combination treatments of AF and pPBS to elucidate the mechanism of action. Since LN-229 and U-87 cell lines were considered sensitive compared to T98G, a lower concentration of 1.5 µM AF was used for sensitive cell lines and a higher concentration of 5 µM AF was used for T98G cells, to examine the combinatorial effects of AF and 25% pPBS. GBM cells were treated with AF for 4 h, followed by 25% pPBS for 4 h. The total GSH levels (Figure 2c) were stable in LN-229 cells and decreased upon statistically significant oxidation of GSH in U-87 and T98G, represented as the GSH/GSSG ratio (Figure 2d), after treatment with AF alone or in combination with 25% pPBS. In U-87 and T98G, sequential combination treatment led to the highest decrease in total GSH and GSH/GSSG ratio when compared to AF/pPBS monotherapy treatments. This indicates that high levels of the available GSH become oxidized after combination therapy of AF and pPBS, resulting in an exhaustion of the GSH system. Higher doses of AF monotherapy (2 µM for LN-229 and U-87; or 7.5 µM for T98G) showed similar effects when compared to the combination therapy. In contrast, increasing the dose of pPBS monotherapy (50% for LN-229 and U-87; or 62.5% for T98G) induced only a minor decrease in total GSH, which was significant in the LN-229 cells (Supplementary Figure S1a,b). 

AF was verified as a TrxR inhibitor, since TrxR activity was fully depleted after 4 h of treatment with 1.5 µM AF as monotherapy or in the treatment in combination with pPBS (Figure 2E). A similar inhibitory effect was also observed with higher dosages of AF (2 µM for LN-229 and U-87; or 7.5 µM for T98G) (Supplementary Figure S1c). pPBS alone (both 25 and 50%; or 62.5% pPBS) showed only a slight decrease in TrxR activity in LN299 cells (Figure 2e and Supplementary Figure S1c). 

AF (both 1.5 or 5 µM; and 2 or 7.5 µM) monotherapy as well as pPBS (25%) monotherapies caused a significant accumulation of intracellular ROS in all GBM cells. This accumulation was significantly more pronounced after 4 h of AF and pPBS combination treatment (Figure 2f). This shows that both therapies enhanced each other for intracellular ROS accumulation. Similar high ROS accumulations were observed when the cells were treated with a higher dose of pPBS (50% for LN-229 and U-87; or 62.5% for T98G), confirming the role of exogenous ROS inducers since minimal or no effects on antioxidant levels were observed (Supplementary Figure S1d). After a prolonged treatment period of 24 h, the intracellular ROS levels reverted to baseline levels in the T98G cell line in both the monotherapies and combination therapy, in contrast to the LN-229 and U-87 cell lines (Supplementary Figure S2a). In line with the stronger baseline GSH levels and TrxR activity, T98G is suggested to have a stronger antioxidant capacity, which explains the resistance towards this ROS-inducing combination therapy. In order to investigate if ROS overproduction was involved in the enhanced cell death induced by the combination of AF and pPBS, N-acetyl cysteine (NAC), a thiol-reducing antioxidant agent, was used to scavenge ROS. The cell death induced by AF and pPBS was completely rescued by NAC pretreatment in all GBM cells, further suggesting the involvement of ROS (Supplementary Figure S2b). Since hydrogen peroxide (H_2_O_2_) was the most abundant, and long-lived ROS present in pPBS treatment, we further evaluated the role of H_2_O_2_ in the killing mechanisms after combination treatment. Addition of catalase, a H_2_O_2_ scavenger, abolished the cytotoxic effect induced by pPBS treatment alone in both LN-229 and U-87 cell lines. However, catalase did not fully suppress the cytotoxic potential when pPBS was combined with AF, or in the case of AF monotreatment, in the U-87 cell line. In the LN-229 cell line, catalase showed stronger inhibition of the killing effect after combination treatment and AF monotreatment when compared to the U-87 cells (Supplementary Figure S2c). 

Together, these data show that AF is a potent inhibitor of TrxR activity in GBM cells, which saturates their GSH systems leading to a modest increase in intracellular ROS levels. While pPBS by itself had limited effect on the TrxR activity and the GSH systems, the inhibition of the antioxidant system by AF significantly increased intracellular ROS accumulation following pPBS treatment. 

### 3.3. The Combination of AF and pPBS Induces Apoptotic and Ferroptotic Characteristics

**Figure 3:**
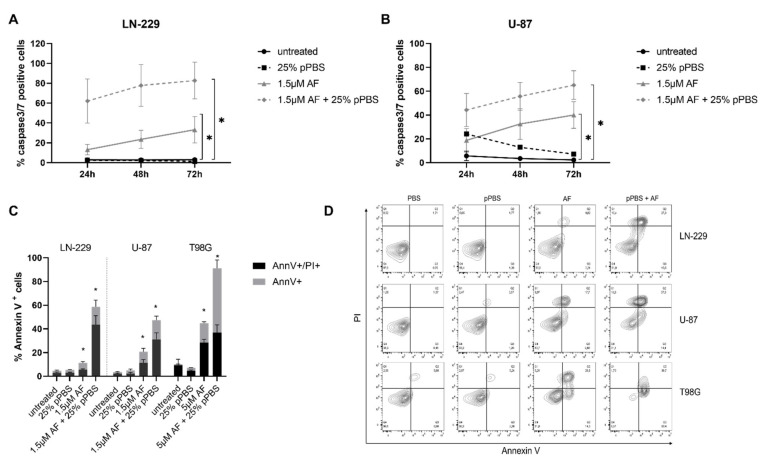
The combination treatment of AF and pPBS induces apoptotic cell death in GBM cell lines. (**A**) Percentage of caspase-3/7 green positive LN-229 cells after 24, 48 and 72 h of treatment. (**B**) Percentage of caspase-3/7 green positive U-87 cells after 24, 48 and 72 h of treatment. (**C**) Percentage of Annexin V+ cells after 48 h of treatment, subdivisions of AnnV+/PI- and AnnV+/PI+ are made. (**D**) Representative contour plots showing the flow cytometric analysis of Annexin V and PI staining after 48 h of treatment. Q1 = AnnV-/PI+, Q2 = AnnV+/PI+, Q3 = AnnV-/PI-, Q3 = AnnV+/PI-. Graphs represent mean ± SEM of ≥3 independent experiments. * *p* ≤ 0.05 denotes a statistically significant difference compared with untreated control.

Next, we unraveled the underlying type of induced cancer cell death after combination treatment of AF and pPBS. In order to investigate the effect of the mono- and combination treatment on cell apoptosis, Annexin V/PI expression and caspase 3/7 activity were determined. A significant time-dependent increase in caspase 3/7 positive U-87 and LN229 cells was observed after combination therapy, which was higher when compared to AF/pPBS monotherapy treatments (Figure 3a,b). Moreover, AF alone induced a significant increase in caspase 3/7 positive cells; however this was to a smaller extent when compared to the combination treatment. 25% pPBS had no effect on caspase 3/7 activity in the LN229 cell line. The U-87 cell line showed a low response in caspase 3/7 activity after 24 h of 25% pPBS treatment. This response disappeared after 72 h of 25% pPBS treatment. T98G cells have green autofluorescence, and therefore are not compatible with the caspase 3/7 reagent, thus they were not considered within this experiment. Additionally, a significant increase of Annexin V+/PI+ and Annexin V+/PI- proportions of apoptotic cells was observed after 48 h of combination treatment of AF and pPBS in all cell lines (Figure 3c,d). Interestingly, the proportion of Annexin V+/PI- cells in T98G was larger when compared to the other cell lines, suggesting that apoptosis was more abundant in the T98G cells with higher concentrations of AF. Unfortunately, we could not validate this with the caspase 3/7 assay due to autofluorescence. 

**Figure 4:**
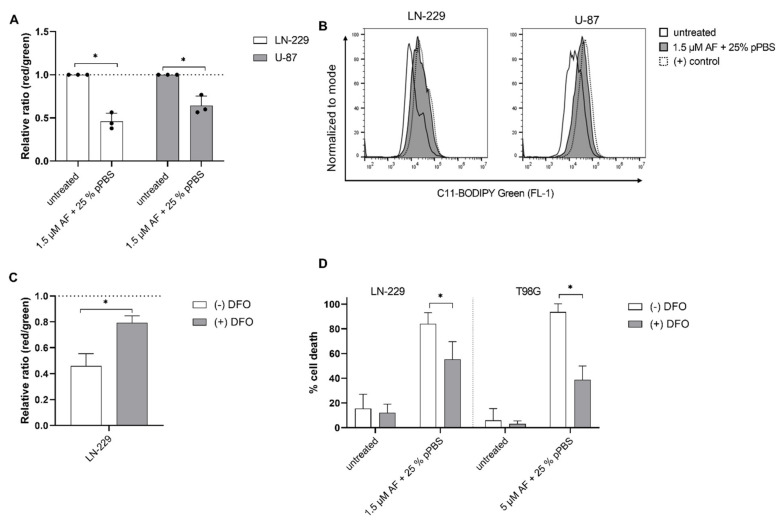
The combination treatment of AF and pPBS induces ferroptotic characteristics in GBM cell lines. (**A**) Lipid peroxidation presented as the relative ratio of red/green MFI signal after a flow cytometric analysis of the C11-BODIPY 581/591 reagent after 48 h of treatment of AF (1.5 µM)) in combination with 25% pPBS. (**B**) Representative overlay histograms of C11 BODIPY green signal (FL-1) after combination treatment of 1.5 µM AF with 25% pPBS for 48 h or cumene hydroperoxide (positive control) for 2 h. (**C**) Lipid peroxidation presented as the relative ratio of red/green MFI signal of the C11-BODIPY 581/591 reagent in absence or in presence of DFO (100 µM) after 48 h of combination therapy with 1.5 µM AF and 25% pPBS in LN-229 cells. (**D**) Percentage of cell death after 48 h of treatment of AF (1.5 µM or 5 µM) in combination with 25% pPBS in the absence and presence of DFO (100 µM), an inhibitor of ferroptosis. Graphs represent mean ± SD of ≥3 independent experiments. * *p* ≤ 0.05 denotes a statistically significant difference compared with untreated control.

ROS are known to interact with lipids leading to lipid peroxidation which can result in ferroptotic cell death. Lipid peroxidation increased significantly after treatment with the combination therapy of AF and pPBS, in the U-87 and LN-229 cell lines (Figure 4a,b). Moreover, deferoxamine (DFO), an inhibitor of lipid peroxidation, was able to inhibit this process in the LN-229 cells (Figure 4c). Again, T98G was excluded in the flow cytometric examination of lipid peroxidation due to its autofluorescence, however, T98G could be included for the cell death analysis after inhibition with DFO. Interestingly, combination-treatment-induced cell death was inhibited by DFO in the LN-229 and T98G cells (Figure 4d). For the U-87 cell line, 100 µM of DFO was shown to be toxic for the cells (Supplementary Figure S3a). A lower concentration of DFO (50 µM) was added to the U-87 cell line to determine the inhibition of cell death and lipid peroxidation after combination therapy. However, no inhibition of cell death or lipid peroxidation was observed in the U-87 cell line (Supplementary Figure S3b,c). The use of a lower concentration of DFO (50 µM) might explain why no inhibition was observed in this cell line. Therefore, no conclusion could be drawn for the U-87 cell line, in the context of DFO inhibition. Another inhibitor, Fer-1 was not able to inhibit the combination-therapy-induced cell death. However, Fer-1 also failed to inhibit therapy-induced lipid peroxidation, which shows that DFO is a more potent inhibitor (Supplementary Figure S4).

Together, these results suggest that the combination of AF and pPBS is able to induce distinct types of cell death, including apoptosis and ferroptosis.

### 3.4. The Combination Treatment of AF and pPBS Induces Immunogenic Cell Death in GBM Cells

**Figure 5:**
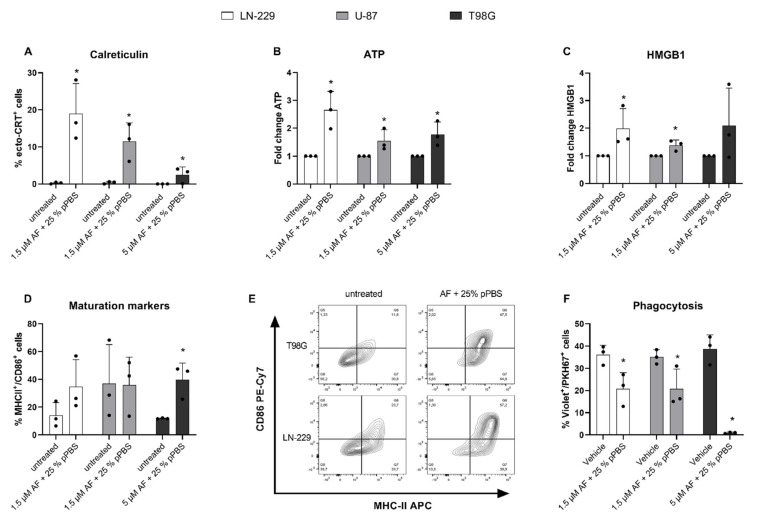
The effect of AF and pPBS combination treatment on the release of ICD danger signals, and DC maturation and phagocytosis. (**A**) Percentage of surface-exposed calreticulin (ecto-CRT) positive cells after 48 h of combination treatment with AF (1.5 µM or 5 µM) and pPBS (25%). (**B**) Secretion of ATP after 4 h of combination treatment with AF (1.5 µM or 5 µM) and pPBS (25%). These data represent the fold change of ATP secretion (nM range). (**C**) Secretion of HMGB1 after 48 h of combination treatment with AF (1.5 µM or 5 µM) and pPBS (25%). These data represent the fold change of ATP secretion (ng/mL range). (**D**) Percentage of MHC-II/CD86 double positive DCs after 48 h of co-culture with AF (1.5 µM or 5 µM) and pPBS (25%) combination-treated LN-229, U-87 or T98G cells (E:T ratio 1:1) using flow cytometry. (**E**) Representative contour plots of the DC population double positive for MHC-II and CD86 in coculture with either PBS-treated or combination-treated T98G (5 µM AF + 25% pPBS) and LN-229 (1.5 µM AF + 25% pPBS). (**F**) Percentage of phagocytosis after 48 h of violet-labeled DC in co-culture with AF (1.5 µM or 5 µM) and pPBS (25%) combination-treated PKH67-labeled GBM cells (E:T ratio 1:1). Phagocytosis of PKH67+ tumor cells by violet-labeled DCs is expressed as %PKH67+violet+ cells within the violet+ DC population. Graphs represent mean ± SD of ≥3 independent experiments. * *p* ≤ 0.05 denotes a statistically significant difference compared with untreated control.

Since different cancer treatments have the capacity to elicit ICD, depending on their ability to produce ROS and cause oxidative stress, we investigated the potential of our sequential combination strategies to elicit ICD. Three important hallmarks of ICD [26,27] were significantly elevated in the U-87 and LN-229 cell lines, following combination treatment with AF and pPBS, which included the surface expression of CRT (ecto-CRT), as well as extracellular ATP and HMGB1 release. In the T98G cell line, only the expression of CRT and the ATP release were significantly elevated after the combination treatment (Figure 5a–c).

Expression and release of these ICD-associated danger signals by dying tumor cells contribute to the activation and maturation of DCs to initiate an effective antitumor immune response. To this end, we investigated if the combination strategy of AF and pPBS could induce DC maturation. We observed a significant increase in the mature CD86+/MHCII+ DC population (Supplementary Figure S5a) after coculture with treated T98G cell lines, whereas only a mild increase was observed in the LN-229 cell line (Figure 5d,e). No increases in DC maturation were observed with U-87 cells. Additionally, we investigated the influence of treated GBM cells on the phagocytotic capacity of immature DCs (Supplementary Figure S4a). The flow cytometric analysis revealed that phagocytosis by immature DCs was significantly inhibited after the treatment of AF in combination with pPBS (Figure 5f). AF was shown to be responsible for this effect, since AF monotherapy also caused inhibition of phagocytosis (Supplementary Figure S5b). 

Collectively, our results show that the combination of AF and pPBS was able to release the most important in vitro hallmarks of ICD in two GBM cell lines as well as induced DC maturation, though to a lesser extent. However, caution should be taken when using AF to induce an effective antitumor immune response since this compound has the capacity to inhibit DC phagocytosis in vitro. 

### 3.5. The Combination Treatment of AF and Plasma Leads to Synergistic Inhibition of 3D Spheroid Growth

**Figure 6:**
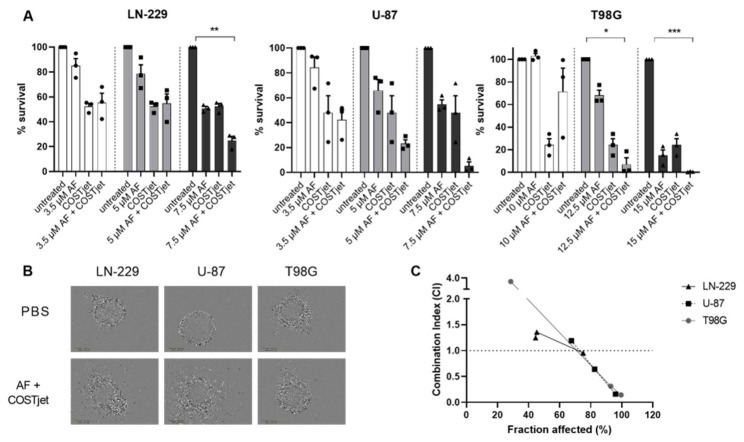
The effect of the sequential combination treatment of AF and plasma on survival of GBM spheroids. (**A**) Dose–response survival curves after 72 h of AF (3.5–15 µM) monotherapy, plasma (COST jet) monotherapy and the combination of AF and plasma. Graphs represent mean ± SEM of ≥3 independent experiments. *p*-values represented as * < 0.05; ** < 0.01; *** < 0.001 denotes a significant interaction between AF and plasma. (**B**) Representative microscopic images after 72 h of untreated and combination-treated GBM cells (7.5 µM AF + COST jet for U-87, LN-229 and 15 µM + COST jet for T98G). (**C**) The corresponding combination index (CI) for each AF concentration. CI > 1 indicates an antagonistic effect, CI = 1 an additive effect, and CI < 1 a synergistic effect.

We then examined the combination strategy in a 3D single-spheroid model, using higher concentrations of AF (3.5 µM–7.5 µM for the sensitive cell lines and 10–15 µM for the resistant cell line). Since indirect pPBS treatment had little or no effect on the viability of the spheroids (Supplementary Figure S6), a direct plasma treatment method was used, which was previously optimized in our lab and described by Privat-Maldonado et al. [22]. After 4 h of AF treatment, the supernatant with the compound was replaced with PBS, followed by a single direct 3 min exposure to plasma and further incubation for 90 min. Afterwards, PBS was replaced by the supernatant, containing AF, of the corresponding spheroid for a total of 72 h. A decrease in cell survival was observed after mono- and combination treatments of AF and plasma (Figure 6a,b). The interaction of AF and plasma was only significant with higher AF concentrations in LN-229 (7.5 µM AF) and T98G (10 and 12.5 µM AF) cell lines, as shown in Figure 6a,b. Additionally, the combination index showed that the effect of AF was synergistically enhanced by plasma, however, this was only in combination with higher AF concentrations (CI < 1 with 7.5 µM for LN-229 and U-87 or 15 µM for T98G). In combination with lower concentrations of AF, the effect on cell survival was shown even to be antagonistic (CI > 1, Figure 6c).

### 3.6. The Combination Treatment of AF and Plasma Leads to Reduced Tumor Growth Kinetics and Prolonged Survival of GBM-Bearing Mice

**Figure 7:**
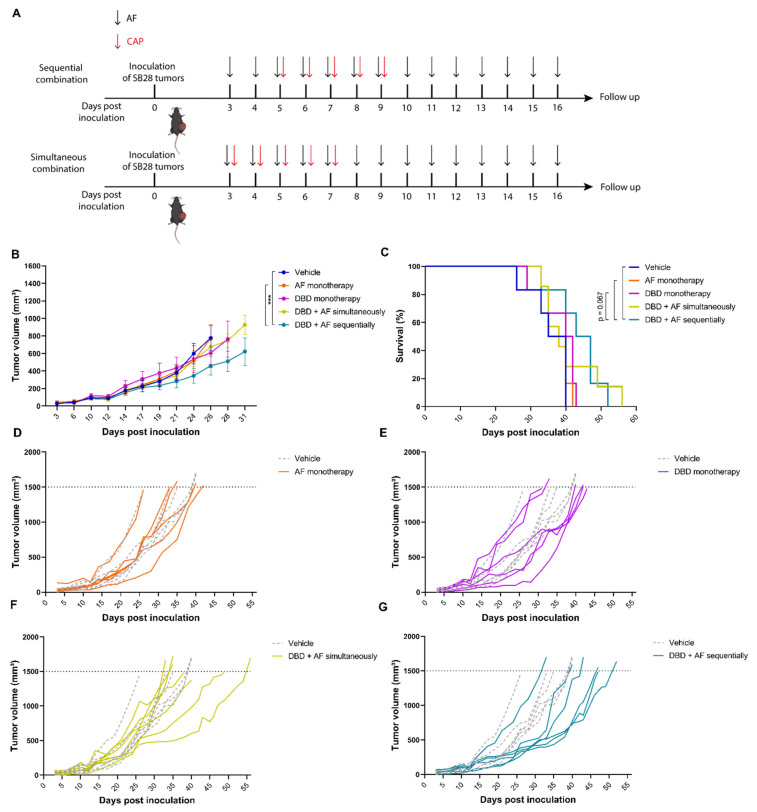
Tumor kinetics and survival after AF and plasma combination therapy. (**A**) Treatment schedule showing timing of AF treatment (15 mg/kg orally administered for 14 consecutive days) with black arrows and cold atmospheric plasma (CAP) treatment (10 s direct application with DBD device for 5 consecutive days) with red arrows. (**B**) Tumor volume kinetics (*n* = 6 or 7 mice per group) after treatments as indicated. Data represent mean ± SEM. (**C**) Survival of SB28 mice (*n* = 6 or 7 mice per group) after treatments as indicated. (**D**–**G**) Spaghetti plots of tumor volumes for individual mice in each treatment group (solid lines) compared to individual untreated mice (dotted lines). * *p* ≤ 0.05, ** *p* ≤ 0.01, *** *p* ≤ 0.001 denotes statistically significant differences.

Since we showed that the combination of AF and plasma induced a synergistic ROS-mediated response in vitro, we further investigated whether the combined treatment of AF and plasma might lead to augmented antitumor response in the SB28 GBM-bearing mouse model. The combination of AF and plasma was delivered in two different treatment schedules, either simultaneously or sequentially (Figure 7a). We demonstrated that the sequential combination regimen resulted in a significantly decreased tumor volume and significantly increased survival of the SB28-bearing mice (Figure 7b–g). When this sequential combination regimen was compared to the single treatments of AF and plasma, there was only a significant difference between the group receiving AF monotherapy in the cases of tumor volume and survival. In the simultaneous combination treatment regimen, there was no statistically significant decrease in tumor volume and no statistically significant increase in survival that was observed when compared to the untreated group and both single treated groups.

## 4. Discussion

Oxidative stress due to elevated ROS levels and an inability to balance the intracellular redox state is considered the ‘Achilles’ heel’ of cancer cells and has recently been highlighted as a promising target for anticancer strategies [4,28]. In this study, we hypothesized that increasing oxidative stress, through a combination strategy of delivering exogenous ROS and inhibiting the protective antioxidant system, could result in a synergistic and promising anti-GBM treatment strategy. Therefore, we used cold atmospheric plasma as a novel anti-cancer therapy to disturb ROS homeostasis in cancer cells via delivery of exogenous ROS, as previously described in different tumor types, including GBM [12,29]. Additionally, AF was selected as inhibitor of the antioxidant defense system through the inhibition of TrxR. The TrxR levels in GBM patients are 31% higher in circulating blood and 5 times higher in GBM tissue when compared to matched controls, both indicating a significant pathophysiological role for TrxR in GBM and emphasizing the therapeutic potential of AF [30]. In this context, AF has already shown to be a promising partner to be combined with temozolomide in a novel treatment strategy for GBM [18,31].

The goal of this study was to study whether AF can be used to sensitize GBM cells to cold atmospheric plasma treatment. Therefore, we tested a sequential treatment regimen of pretreatment with AF, combined with plasma treatment, in vitro. This sequential scheme was chosen based on the hypothesis that AF could inhibit cellular antioxidant systems to reduce the scavenging of ROS which were directly added by plasma treatment. Additionally, we confirmed this hypothesis in vivo, since the sequential combination of AF prior to direct plasma treatment was better when compared to simultaneous combination treatment in reducing tumor kinetics and increasing survival. 

Our results indicate that pPBS synergistically enhances the therapeutic effect of AF in three different GBM cell lines, thereby confirming our hypothesis. The synergistic effects of AF and plasma were further investigated in vitro in 3D tumor spheroids. This spheroid model is characterized with the biophysical properties of solid tumors, such as oxygen and nutrient gradients, which are relevant when investigating oxidative-stress-inducing treatment strategies [32]. For the treatment of 3D spheroids, we used a different plasma device, which has already been shown to be effective when directly treating GBM spheroids [22]. This direct plasma treatment method generates a higher concentration of ROS, which we showed to be necessary for effectivity in spheroids. Synergistic effects between AF and pPBS or AF and direct plasma treatment were observed in 2D cell cultures and 3D spheroids, respectively. Interestingly, with lower AF concentrations the combinatorial effect was shown to be antagonistic in 3D spheroids. We hypothesize that in combination with lower concentrations of AF, the accumulation of intracellular ROS was too small, causing GBM spheroids to adapt by enhancing their protective antioxidant system. Indeed, it has been reported that radiation-induced oxidative stress was considerably less in spheroids as compared to monolayers, and this corresponded with an increase in radio-resistance, due to alterations in intracellular ROS levels and redox status (e.g., activity of antioxidant enzymes) during the spheroid development [33,34]. In order to be effective in vivo, it might be that high dosages of AF are required to treat cancer, even in combination with other therapies [35,36].

Differences in sensitivity were observed between the different GBM cell lines, with T98G being more resistant when compared to LN-229 and U-87 cell lines. This could be explained by higher GSH baseline levels and TrxR activity, and corresponding lower ROS levels, in the resistant T98G cell line. Consistently, it was shown that T98G was more resistant to temozolomide chemotherapy as a result of lower ROS levels as well as higher total antioxidant capacity and GSH concentration [37]. Therefore, increasing exogenous ROS levels together with inhibition of the antioxidant defense system could overcome this therapy resistance. 

The role of ROS was investigated to elucidate the underlying mechanisms of cell death after mono- and combination treatments. We demonstrated a high intracellular ROS accumulation after treatment with the combination of AF and pPBS. We confirmed this ROS-mediated response in vitro, as the ROS scavenger NAC reversed the combination-treatment-mediated cell death in all GBM cell lines. However, catalase only rescued the pPBS-induced cell death, revealing that exogenous H_2_O_2_ was the primary mediator of the pPBS-induced cell death, as previously described [38,39]. In the LN-229 cell line, catalase also showed a strong inhibition after both the combination treatment and AF monotreatment. Such an inhibition was less pronounced in the U-87 cell line after the combination treatment or the AF monotreatment, which indicated H_2_O_2_-independent effects which contributed to the killing capacity after AF treatment alone and in combination with pPBS. In addition to catalase, the Trx/TrxR and GSH antioxidant systems are partly responsible for the removal of endogenous H_2_O_2_. Besides H_2_O_2_, the Trx and GSH systems participate in the reduction of different kinds of endogenous ROS and RNS [40]. Since AF was shown to inhibit these two antioxidant systems, the incomplete removal of H_2_O_2_ and other types of ROS might explain why catalase did not completely abolish AF- and combination-induced cell death. 

Accumulation of intrinsic ROS has shown to be important, not only for the induction of apoptosis, but also ferroptosis [41]. Therefore, we did a more in-depth analysis of the underlying ROS-mediated cell death mechanism after treatment with AF and pPBS. We discovered that this ROS-inducing combination treatment sensitized the GBM cells for caspase-3/7-dependent apoptosis, based on an increase of caspase-3/7- and Annexin V-positive cells, as well as ferroptosis, due to lipid peroxidation and cell death inhibition by an iron chelating agent, DFO. Contrary to DFO, the level of protection against cell death by Fer-1 was incomplete, as Fer-1 failed to protect cells from lipid peroxidation. Previously, we already showed that DFO and Fer-1 were able to partially prevent cell death in non-small cell lung cancer after AF treatment alone [42]. Excessive ROS, induced after combination treatment of AF and pPBS, could explain this incomplete protection of Fer-1 [43]. To date, previous studies have reported that AF and pPBS induce cancer cell death through ROS-mediated endoplasmic reticulum (ER) stress and activation of the apoptotic pathway in different cancer types [39,44,45,46,47]. Other findings indicate that both treatments are linked to ferroptosis which is triggered by ROS accumulation, leading to iron-mediated lipid peroxidation and cell death [39,48]. Recently, our research group also demonstrated the induction of ferroptosis and apoptosis in non-small cell lung cancer after AF treatment [42]. However, we are the first to show that the combination of both treatment types sensitizes GBM cells for apoptotic and ferroptotic cell death. 

Furthermore, we demonstrated the immunogenic potential of the AF and pPBS combination-treatment-induced apoptotic and ferroptotic cell death in GBM cells. Besides the immunogenic potential of apoptosis, it was recently described that ferroptosis is a novel approach for the induction of antitumor immunity which is triggered by ferroptosis-dependent ICD [49,50]. It has been suggested that cancer cells that die through distinct ICD-inducing mechanisms might achieve a superior antitumor immune response [49]. The induction of oxidative stress through the production of ROS is the common underlying factor in different ICD-inducing therapies [51]. Since the induced cytotoxic effect in our study was clearly dependent on the high accumulation of intracellular ROS, we investigated if the combination of AF and pPBS was able to elicit different ICD-related DAMPs, and subsequently stimulate maturation and phagocytosis by DCs. In a previous study, we already showed the release of several ICD markers and subsequent maturation of DCs and phagocytosis by DCs, after pPBS treatment in pancreatic cancer cells [12]. Here, we also reported a significant release of danger signals and the maturation of DCs after the combination treatment of AF and pPBS in a GBM cell panel. However, the phagocytotic capacity of DCs was inhibited after combination treatment. A similar inhibition of phagocytosis was observed following AF monotherapy, suggesting that AF was responsible for the effect. This is in line with other studies showing that AF could inhibit phagocytosis [52]. This can be explained by the history of its use as an anti-rheumatoid arthritis drug which is linked to the inhibition of the pro-inflammatory mediators and oxidative burst in monocytes and granulocytes that are necessary for effective phagocytosis [53]. 

In line with the in vitro data, we showed that the sequential combination regimen of AF and cold atmospheric plasma induced a decrease in tumor volume and an increase in the survival of the SB28 GBM-bearing mouse model, which was significantly better than both the untreated control and the AF single-agent treatment group. This was in contrast with the simultaneous combination regimen, which showed no significant effect on tumor volume and survival. These results are in line with our in vitro data, indicating that it is important to inhibit the endogenous defense system before adding excessive amounts of exogenous ROS. A limitation of the present study may be that the mouse models do not completely mimic the human situation since the tumor cells were injected subcutaneously. Nevertheless, we were able to prove our hypothesis: that inhibition of the antioxidant system by AF was able to sensitize GBM tumors to direct plasma treatment in an in vivo setting.

Together, these in vitro and in vivo results highlight the therapeutic value of combining AF with cold atmospheric plasma treatment in GBM. 

## 5. Conclusions

Altogether, the effectiveness of the combination treatment of AF and plasma to synergistically eradicate GBM cells in vitro was shown through different ROS-dependent molecular mechanisms, those being apoptosis and ferroptosis. Both cell-death mechanisms resulted in a significant increase of DAMPs and the maturation of DCs in vitro, indicating the potential antitumoral immunogenic effect. Contrary to these immunostimulatory effects, a potential inhibitory effect on the phagocytotic capacity of DCs due to AF should be taken into consideration for future research when exploring combination strategies with AF. Importantly, AF in combination with cold atmospheric plasma effectively reduced tumor growth and prolonged survival in vivo. In conclusion, our study provides a novel therapeutic strategy for GBM to enhance the efficacy of oxidative-stress-inducing therapy through a combination of increasing exogenous ROS and inhibiting the protective antioxidant system.

## Figures and Tables

**Table 1:** Cell death, cell inhibition and synergism of AF and pPBS combination treatment of GBM cell lines.

(A) Cell Inhibition and Treatment Synergism			
Treatment	LN-229		
	IC50	*p*-value	CI (1.5 µM AF + 25% pPBS)
*AF*	0.762 (±0.034)	/	/
*AF + 25% pPBS*	0.350 (±0.064)	<0.0001	0.611 (±0.214)
	**U-87**		
	**IC50**	***p*-value**	**CI (1.5 µM AF + 25% pPBS)**
*AF*	0.455 (±0.088)	/	/
*AF + 25% pPBS*	0.183 (±0.0.092)	<0.0001	0.6211 (±0.227)
	**T98G**		
	**IC50**	***p*-value**	**CI (5 µM AF + 25% pPBS)**
*AF*	2.364 (±0.234)	/	/
*AF + 25% pPBS*	1.599 (±0.239)	0.8865	0.687 (±0.074)
**(B) Cell death and treatment synergism**			
**Treatment**	**LN-229**		
	**EC50**	***p*-value**	**CI (1.5 µM AF + 25% pPBS)**
*AF*	2.335 (±0.142)	/	/
*AF + 25% pPBS*	1.037 (±0.065)	<0.0001	0.460 (±0.155)
	**U-87**		
	**EC50**	***p*-value**	**CI (1.5 µM AF + 25% pPBS)**
*AF*	1.739 (±0.117)	/	/
*AF + 25% pPBS*	0.755 (±0.098)	0.00015	0.506 (±0.105)
	**T98G**		
	**EC50**	***p*-value**	**CI (5 µM AF + 25% pPBS)**
*AF*	7.395 (±0.269)	/	/
*AF + 25% pPBS*	4.487 (±0.078)	<0.0001	0.446 (±0.077)

(**A**) Cell inhibition and synergism of AF and pPBS combination treatment. The table gives an overview of the IC50 values (±SE) of AF after AF monotherapy (normalized to PBS) and treatment in combination with 25% pPBS (normalized to 25% pPBS) for each cell line. IC50 values represent the concentration of AF where the survival response is reduced by half. The average combination index (CI ± SEM) based on cell inhibition is provided for one concentration of AF (1.5 µM for LN-229 and U-87, and 5 µM for T98G) for the combination therapy. (**B**) Cell death and synergism of AF and pPBS combination treatment. The table gives an overview of the EC50 values (±SE) of AF after AF monotherapy and treatment in combination with 25% pPBS for each cell line. EC50 values represent the concentration of AF that gives a half-maximal cytotoxic response. The average combination index (CI ± SEM), based on cell death response, is provided for one concentration of AF (1.5 µM for LN-229 and U-87, and 5 µM for T98G) for the combination treatment. CI > 1 indicates an antagonistic effect, CI = 1 an additive effect and CI < 1 a synergistic effect.

## Data Availability

Not applicable.

## Supplementary Materials

The following are available online at www.mdpi.com/2073-4409/10/11/2936/s1, Figure S1: The effect of monotherapies of AF or pPBS on protein targets related to the antioxidant defense system., Figure S2: The effect on intracellular ROS levels after combination treatment of AF and pPBS, Figure S3: The effect of DFO on U-87 cell line, Figure S4: The effect of ferrostatin-1 on cell death and lipid peroxidation induced via combination treatment of AF and pPBS in GBM cell lines., Figure S5: Gating strategy and effect of monotherapies on phagocytosis of GBM cells, Figure S6: The effect of pPBS and AF on survival of GBM spheroids.
