# Supplementary material for: Auranofin and Cold Atmospheric Plasma Synergize to Trigger Distinct Cell Death Mechanisms and Immunogenic Responses in Glioblastoma
Source: Cells. 2021 Oct 28;10(11):2936. doi: 10.3390/cells10112936 (PMC8616410; doi:10.3390/cells10112936)
Supplement: Supplementary file 1 [file cells-10-02936-s001.zip › cells-1404189-supplementary.pdf]

Supplementary Files

Auranofin and cold atmospheric plasma synergize to trigger distinct cell death mechanisms and immunogenic responses in glioblastoma

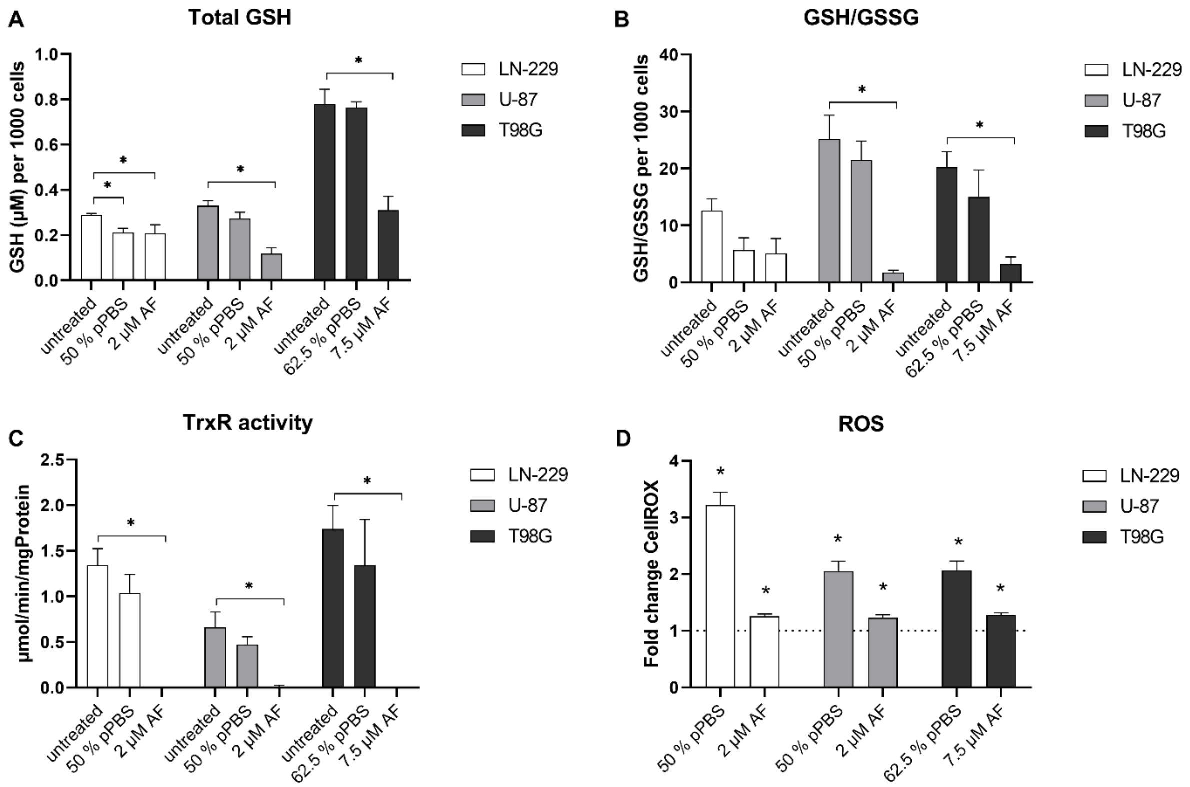

Supplementary Figure S1. The effect of monotherapies of AF or pPBS on protein targets related to the antioxidant defense system. (A) GSH protein levels per 1000 cells after 4 hours of treatment with monotherapies of AF (2 µM or 7.5 µM) and 50% pPBS. (B) Ratio GSH/GSSG per 1000 cells after 4 hours of treatment with monotherapies of AF (2 µM or 7.5 µM) and 50% pPBS. (C) TrxR activity after 4 hours of treatment with monotherapies of AF (2 µM or 7.5 µM) and 50% pPBS. (D) Intracellular ROS levels shown as fold change of CellROX Green Calibration Units (GCU) for U-87 and LN-229 and CellROX Red Calibration Units (RCU) for T98G cells, relative towards untreated after 4 hours of treatment with monotherapies of AF (2 µM or 7.5 µM) and 50% pPBS. Graphs represent mean ± SEM of ≥ 3 independent experiments. *$p \leq 0.05$ denotes statistically significant difference compared with untreated control.

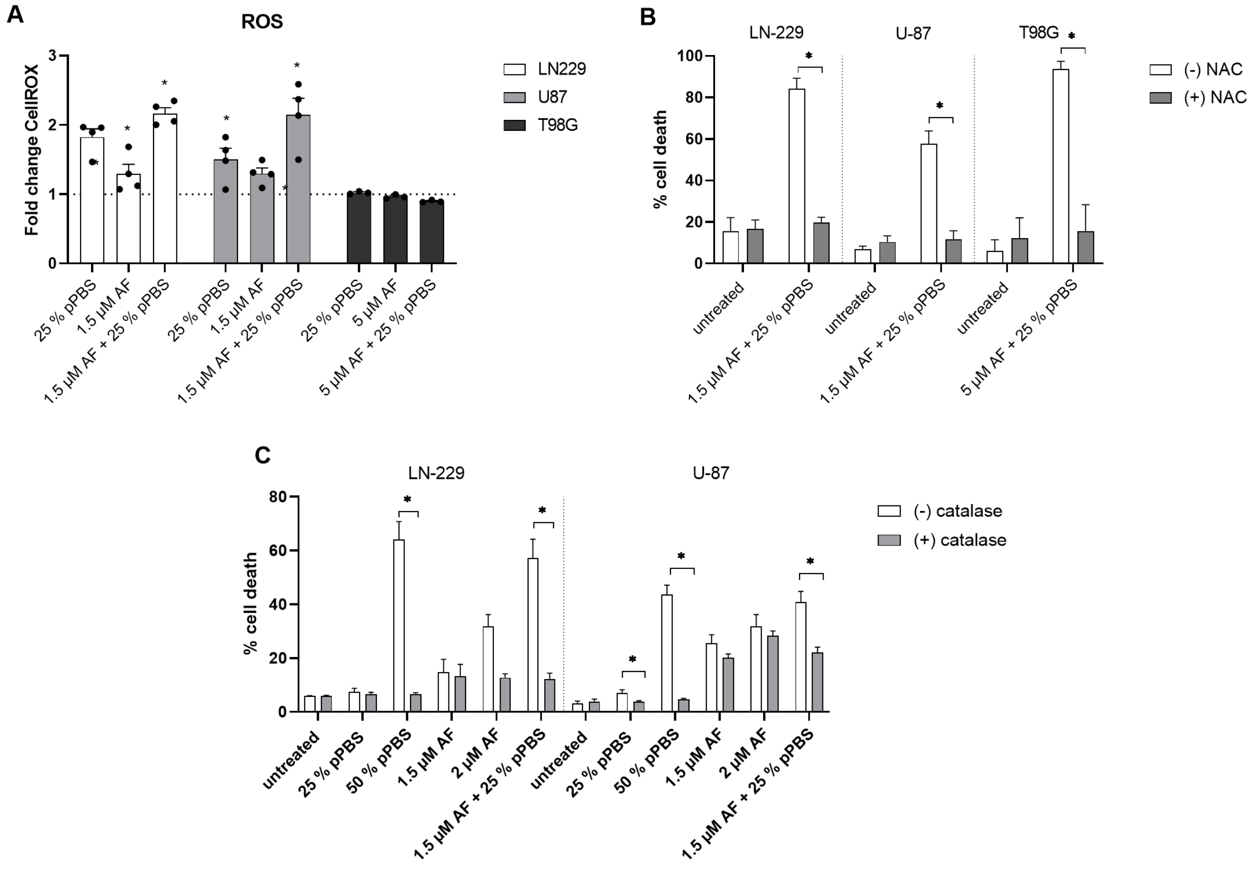

Supplementary Figure S2. The effect on intracellular ROS levels after combination treatment of AF and pPBS. (A) Intracellular ROS levels are shown as fold change of CellROX Green Calibration Units (GCU) for U-87 and LN-229 and CellROX Red Calibration Units (RCU) for T98G cells, relative towards untreated cells after 24 hours of treatment with monotherapies or combination treatment of AF (1.5 µM or 5 µM) and 25% pPBS. *p ≤ 0.05 denotes statistically significant difference compared with untreated control. (B) Percentage of cell death in the absence or presence of NAC pretreatment, after 48 hours of treatment with combination of AF and pPBS. (C) Percentage of cell death in the absence or presence of catalase pretreatment, after 48 hours of treatment. Graphs represent mean ± SEM of ≥ 3 independent experiments. *p ≤ 0.05 denotes statistically significant difference.

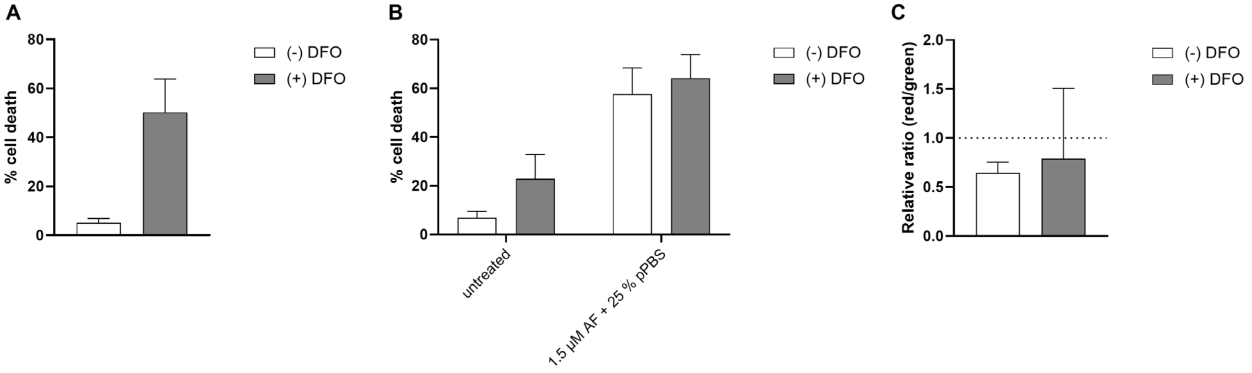

Supplementary Figure S3. The effect of DFO on U-87 cell line. (A) Percentage of cell death in untreated U-87 cells in the absence and presence of 100 μM DFO. (B) Percentage of cell death after 48 hours of treatment of 1.5 μM AF in combination with 25% pPBS in the absence and presence of DFO (50 μM). (C) Lipid peroxidation presented as relative ratio of red/green MFI signal of the C11-BODIPY 581/591 reagent in absence or in presence of DFO (50 μM) after combination treatment of 1.5 μM AF and 25% pPBS.

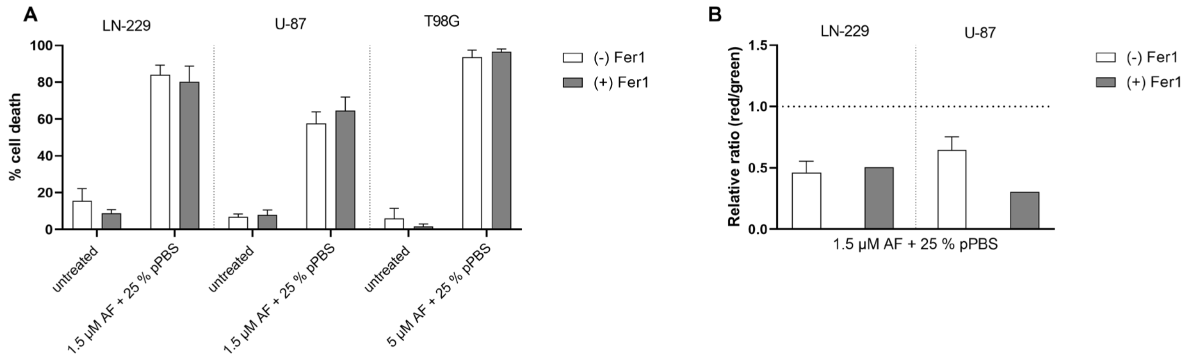

Supplementary Figure S4. The effect of ferrostatin-1 on cell death and lipid peroxidation induced via combination treatment of AF and pPBS in GBM cell lines. (A) Percentage of cell death after 48 hours of treatment of AF (1.5 μM or 5 μM) in combination with 25% pPBS in the absence and presence of Fer-1 (1 μM), an inhibitor of ferroptosis. Graphs represent mean ± SEM of ≥ 3 independent experiments. (B) Lipid peroxidation presented as relative ratio of red/green MFI signal of the C11-BODIPY 581/591 reagent in absence or in presence of Fer-1 (1 μM) after 48 hours of combination therapy with 1.5 μM AF and 25% pPBS.

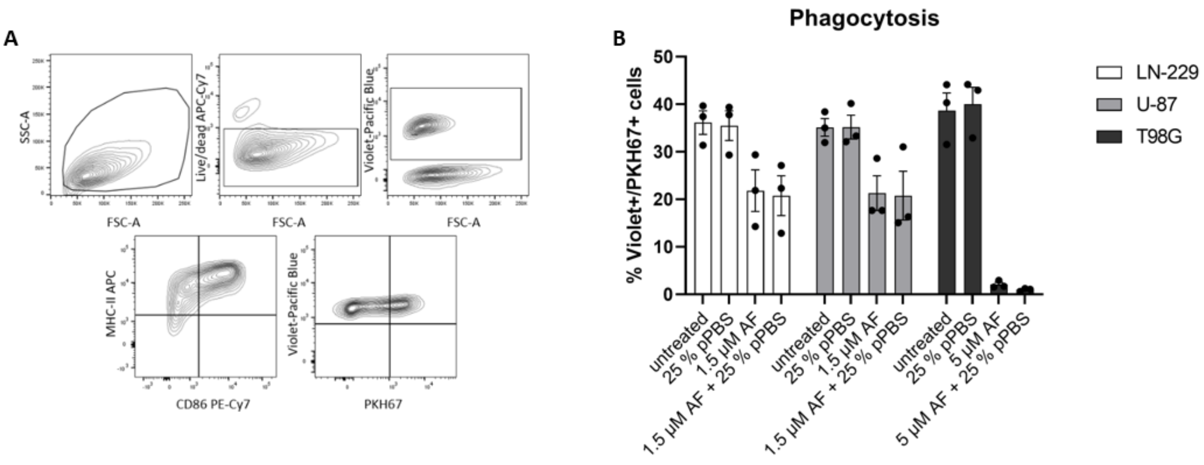

Supplementary Figure S5. Gating strategy and effect of monotherapies on phagocytosis of GBM cells. (A) Gating strategy of maturation of DCs and phagocytosis by DCs. (B) Percentage of phagocytosis after 48 hours of violet-labeled DCs in co-culture with PKH67-labeld GBM cells (E:T ratio 1:1) after treatment with monotherapies and combination therapy of AF (1.5 μM or 5 μM) and pPBS (25%). Phagocytosis of PKH67+ tumor cells by violet-labeled DCs is expressed as %PKH67+violet+ cells within the violet+ DC population. Graphs represent mean ± SEM of ≥ 3 independent experiments. *p ≤ 0.05 denotes statistically significant difference compared with untreated control.

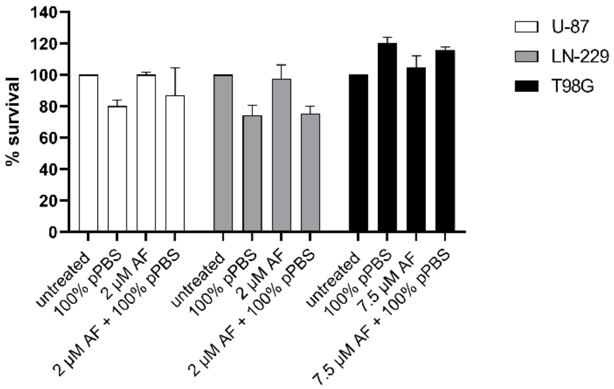

Supplementary Figure S6. The effect of pPBS and AF on survival of GBM spheroids. Dose-response survival curves after 72 hours of AF (2 or 7.5µM) and pPBS (100%) monotreatment and combination treatment. Graphs represent mean ± SEM of ≥ 3 independent experiments. No statistical significant interaction between AF and pPBS was observed.
